# The Role of CHI3L1 (Chitinase-3-Like-1) in the Pathogenesis of Infections in Burns in a Mouse Model

**DOI:** 10.1371/journal.pone.0140440

**Published:** 2015-11-03

**Authors:** Stefan Bohr, Suraj J. Patel, Radovan Vasko, Keyue Shen, Alexander Golberg, Francois Berthiaume, Martin L. Yarmush

**Affiliations:** 1 Center for Engineering in Medicine, Shriners Hospitals for Children and Department of Surgery, Massachusetts General Hospital, Harvard Medical School, Boston, MA, United States of America; 2 Department of Medicine, New York Medical College, Valhalla, NY, United States of America; 3 Department of Biomedical Engineering, Rutgers University, New Brunswick, NJ, United States of America; 4 Department Plastic and Hand Surgery—Burn Center, UKA University Clinics RWTH, Aachen, Germany; 5 Department of Nephrology & Rheumatology, UMG University Clinics, Goettingen, Germany; 6 Porter School of Environmental Studies, Tel Aviv University, Tel Aviv, Israel; University of Tübingen, GERMANY

## Abstract

In severe burn injury the unique setting of a depleted, dysfunctional immune system along with a loss of barrier function commonly results in opportunistic infections that eventually proof fatal. Unfortunately, the dynamic sequence of bacterial contamination, colonization and eventually septic invasion with bacteria such as Pseudomonas species is still poorly understood although a limiting factor in clinical decision making. Increasing evidence supports the notion that inhibition of bacterial translocation into the wound site may be an effective alternative to prevent infection. In this context we investigated the role of the mammalian Chitinase-3-Like-1 (CHI3L1) non-enyzmatic protein predominately expressed on epithelial as well as innate immune cells as a potential bacterial-translocation-mediating factor. We show a strong trend that a modulation of chitinase expression is likely to be effective in reducing mortality rates in a mouse model of burn injury with superinfection with the opportunistic PA14 Pseudomonas strain, thus demonstrating possible clinical leverage.

## Introduction

Opportunistic bacterial infections such as with Pseudomonas (PS), serratia, or acinetobacter species are a common cause of lethality associated with burn injury [1]. The survival of burn patients often not only depends upon the severity, scored by e.g. (i) burn depth, (ii) surface area, (iii) location of the burn but also upon the dynamics of seemingly unavoidable contamination, colonization but preventable opportunistic bacterial invasion [2]. The term ‘opportunistic’ can be misleading but usually refers to microbes which (i) although ubiquitous with the environment rarely cause infections with immunity intact, (ii) wield a natural drug-resistance, (iii) form biofilms [3] and (iv) secrete exotoxins along with invasiveness through not well understood mechanism of *quorum sensing* [4]. PS fulfills all of these criteria [5].

Patients with relevant burn injury typically demonstrate the appearance of positive opportunistic swabs as early as 24 hours post burn. Strong evidence can be found in the literature that the most likely source of contamination e.g. with PS is the individual’s own enteric or pulmonary system [6–8].

A large body of literature is available related to (i) impaired innate immunity function [9–13], (ii), enteric/epithelial interactions with microbes [14, 15], (iii) environmental sensing of microbes [4] or (iv) development of multi-drug-resistance [16] in relation to an overwhelming systemic inflammatory response seen with septicemia or burn injury.

In this context, Chitinase-3-like-1 (CHI3L1, YKL-40 or HC-gp39), has been evaluated as an inflammation-associated inducible molecule which enhances bacterial adhesion and invasion on/into epithelial cells and macrophages [17–24]. CHI3L1 binds to chitin (an N-acetyl glucosamine polysaccharide) with high affinity, but has no apparent enzymatic activity, again suggesting other relevant biological functions apart from enteric enzymatic degradation. Chitin [25] is abundantly found in nature (e.g., fungi, insects, nematodes) but is completely absent in mammals. Nevertheless, mammalian chitinases are naturally found in the skin, intestine, joints, and the lungs of mice and humans [26, 27]. Although bacteria do not express chitin, some bacterial strains such as PS constitutively express chitin-binding proteins (CBPs) [28–31]. It has been suggested that CHI3L1 on epithelial cells enhances adhesion and invasion of CBP-expressing bacteria with potentially high virulence [14, 18, 19, 22]. Specifically, CBP21 expressed by serratia marcescens has been shown to specifically bind CHI3L1 expressed on colon epithelial cells in mice [20]. PS actively produces chitin-binding protein (cbpD) and should possess the ability to directly interact with CHI3L1 as well as other mammalian chitinases via its chitin-binding domain (or motif). This study was designed to investigate this novel aspect in the pathogenesis of infection in burns, and the effect of modulating CHI3L1 expression in the context of severe burn injury in a mouse model.

## Methods

### Experimental mice & animal models

C57BL/6 wild type (WT) mice, >6 weeks old, 20–25 grams of body weight, were obtained from The Jackson Laboratory (Bar Harbor, ME). Animals were housed under standard conditions in an institutional animal facility in accordance with *National Research Council* guidelines and with approval by the *Subcommittee on Research Animal Care* at Massachusetts General Hospital, Boston, MA. Animals were anesthetized by intraperitoneal injection of ketamine/xylazine. For tissue harvest, animals were euthanized by intraperitoneal sodium pentobarbital overdose or carbon dioxide inhalation.

The following models of burn injury as well as an associated opportunistic infection with Pseudomonas species leading to a septicemia-induced systemic inflammatory response (SIR) were used: (1) a full-thickness dorsal scald burn of ~20% body-surface-area (BSA) including a volume resuscitation and pain management protocol as previously described [32]; (2) SIR-induction using i.p. injection of lipopolysaccharides (LPS) from Pseudomonas aeruginosa 10 (Sigma-Aldrich, MO; #L9143-25MG); (3) opportunistic infection of surgically debrided burn wounds (see model 1) with topical application of the wild type PA14 Pseudomonas strain (see also S2 Fig); (4) burn injury-induced entero-hematogenic bacterial translocation (see also S3 Fig) following 48h pre-burn enteric PA14 colonization using gavage-inoculation technique as previously described [33]. Induction of mammalian chitinases prior to burn injury was performed either by s.c. injection of chitosan (Sigma-Aldrich, MO; #448869) preparations or supplementation of the later to drinking water. For in vivo blockage experiments we used sheep anti-mCHI3L1 http://www.rndsystems.com/Products/BAF2649).

Survival assessment was conducted under close surveillance in intervals of ≤ 6h of animal behavior as well as under pain management [buprenorphine: 0.1 mg/kg/50μl saline sc initially and every 12h] as previously described [32]. An animal was a non-survivor if found in a ‘moribund condition’ based on a score sheet system (e.g. loss of grooming, feeding, general activity, hunched position, shivering, dry eyes, apathy) as previously described [34], or already dead (although this was a rare occurrence). Moribund animals were euthanized using pentobarbital overdose (100 mg/kg ip).

### Pseudomonas wild type strain (PA14)

PA14 Pseudomonas strain stock (UCBPPPA14; provided by Department of Microbiology and Molecular Genetics, Harvard Medical School, Boston, MA 02115, USA [35]) was maintained using selective cetrimide agar (Sigma-Aldrich, MO; #70887) and expanded in LB broth (Lennox) media (Sigma-Aldrich, MO; #L3022) at 37°C supplemented with chitin (Sigma-Aldrich, MO; # C7170) or chitosan (see above). For animal experiments, expanded bacterial cultures were washed 5x with and stored in 0.9% saline at 4°C for <2 weeks. Bacterial dosage/load was determined performing agar-plate based dilution-series.

### CFU-counts

For the estimation of colony-forming-units of PA14 Pseudomonas strain, dilution series (1:1 to 1:10^4^) of given samples, e.g. LB media or blood culture, were plated on cetrimide agar plates and incubated at 37°C for 24h as previously described [18].

### Peritoneal lavage

For the evaluation of PA14 Pseudomonas strain enteric translocation, peritoneal lavage was performed using 1ml of 0.9% saline for i.p. injection followed by a white cell count in peritoneal aspirates as previously described [36].

### Immunohistochemistry (IHC)

Histological evaluation of tissues was performed on 10 μm sections of formalin fixed and paraffin embedded samples followed by IHC using standard protocols (www.ihcworld.com). IHC for mCHI3L1 was performed using the following antibodies and detection reagents: sheep anti-mCHI3L1 (R&D Systems, Inc., MN; #BAF2649), normal sheep IgG biotinylated control (R&D Systems, Inc., MN; #BAF020), Vectastain ABC Kit/DAB Peroxidase Substrate Kit (Vectorlabs., CA; #PK-4000, # SK-4100) and hematoxylin nuclear counterstain (Sigma-Aldrich, MO; #HHS16). Images were taken using a Nikon Eclipse E600^™^ microscope (Nikon, Japan) mounted with a Spot PCI-CE^™^ camera (Carl-Zeiss, Germany).

### Gene expression analysis

RNA was extracted from cells or tissue samples using either *TRIzol* ^®^ *Reagent* extraction (Invitrogen, CA; # AM9738) or *AllPrep DNA/RNA/Protein Mini Kit* ^®^ (www.qiagen.com). Total RNA quality was assessed by spectroscopy (NanoDropND-1000, Thermofisher, MA) and microfluidic gel analysis (Agilent RNA 6000 Nano Chip /Agilent^®^2100 Bioanalyzer; Germany) followed by cDNA conversion using the *SuperScript* ^™^ *III Platinum* ^®^ *Two-Step qRT-PCR Kit* (Invitrogen, CA; #11734–068) or a *RT² First Stand Kit* ^®^ *C-03* (Sabiosciences, Germany; #330401) and a *Perkin Etus Thermal Cycler 480* (PerkinElmer, CA). The gene expression patterns were assessed by quantitative PCR (qPCR) with a *RT² SYBR* ^®^ *Green/Rox qPCR Master Mix* (Sabiosciences, Germany; #330523) using a *Stratagene* ^®^ *mx3005P* system (Agilent, Germany). A melting curve was used to confirm the specificity of each primer pair. Each sample was run in triplicate to exclude outliers. Gene expression was analyzed using the ΔΔCT-method (*RT²qPCR Primer Assay User Manual*, *Version2*.*17*, www.sabiosciences.com *)* with GAPDH as the normalizing gene. The average gene expression was computed for each experimental condition (n≥3) relative to the control condition (n≥3). GAPDH primer was purchased (Sabiosciences/Quiagen, Germany; # PPM02946E-200). The following primers were designed using *Primer3 v*. *0*.*4*.*0* software (//frodo.wi.mit.edu/primer3) utilizing the following DNA coding sequences (www.ncbi.nlm.nih.gov/nuccore): mCHI3L1 (as quoted: [18]), F: 5’-GTACAAGCTGGTCTGCTACT, R: 5’-GTTGGAGGCAATCTCGGAAA; mCHIT1 (Gene ID: 71884), F: 5’- AGGTGCCTTACGCCTTCC, R’: 5’- GCCGTAGTGTCCGGATGA; mCHIA (Gene ID: 81600), F: 5’-CCTGCTGGGCCCTATACC, R’: 5’-CCCAGATCATGGCACCTC; PA14-CbpD (Gene ID: WP003140848), F: 5’-GCACTCCTGCCACTCACC, R’: 5’-CGAGGAAGCAGCCGTAGA; PA14-toxA (Gene ID: WP003138083), F: 5’-CGAGGAAGCCTTCGACCT, R’: 5’-GTTGTCGATGGCCAGCTT; PA14-AlgF (Gene ID: WP003140553), F: 5’-CGGTGACGTACAGGCAGA, R’: 5’-CAACCCGGTGAAGGTGAA; PA14-Hagl (Gene ID: WP011666523), F: 5’-GACGGCCTGAGTCTGGA, R’: 5’-AGGCTGCCTTCGCTGAC; PA14-RhlR (syn. PA14-UCBPP) (Gene ID: WP003119559), F: 5’-CGGTTTGCGTAGCGAGAT, R’: 5’-CCAGGCCTTGGGATAGGT;

### Statistics

The results are presented as mean ± SD and/or SEM. Pair wise comparisons were performed using a 2-tailed student’s t test, whereas multiple comparisons were performed by 2-way analysis of variance (ANOVA). The Kaplan–Meier method was used to estimate survival rates of animal groups. Equivalences of the survival curves were tested by log-rank statistics. All analyses were performed using Prism^®^4.0 (GraphPad Software Inc., CA) software. A 2-tailed P< 0.05 was considered statistically significant.

## Results

### Evaluation of chitinase expression in wild type (WT) mice

**Fig 1:**
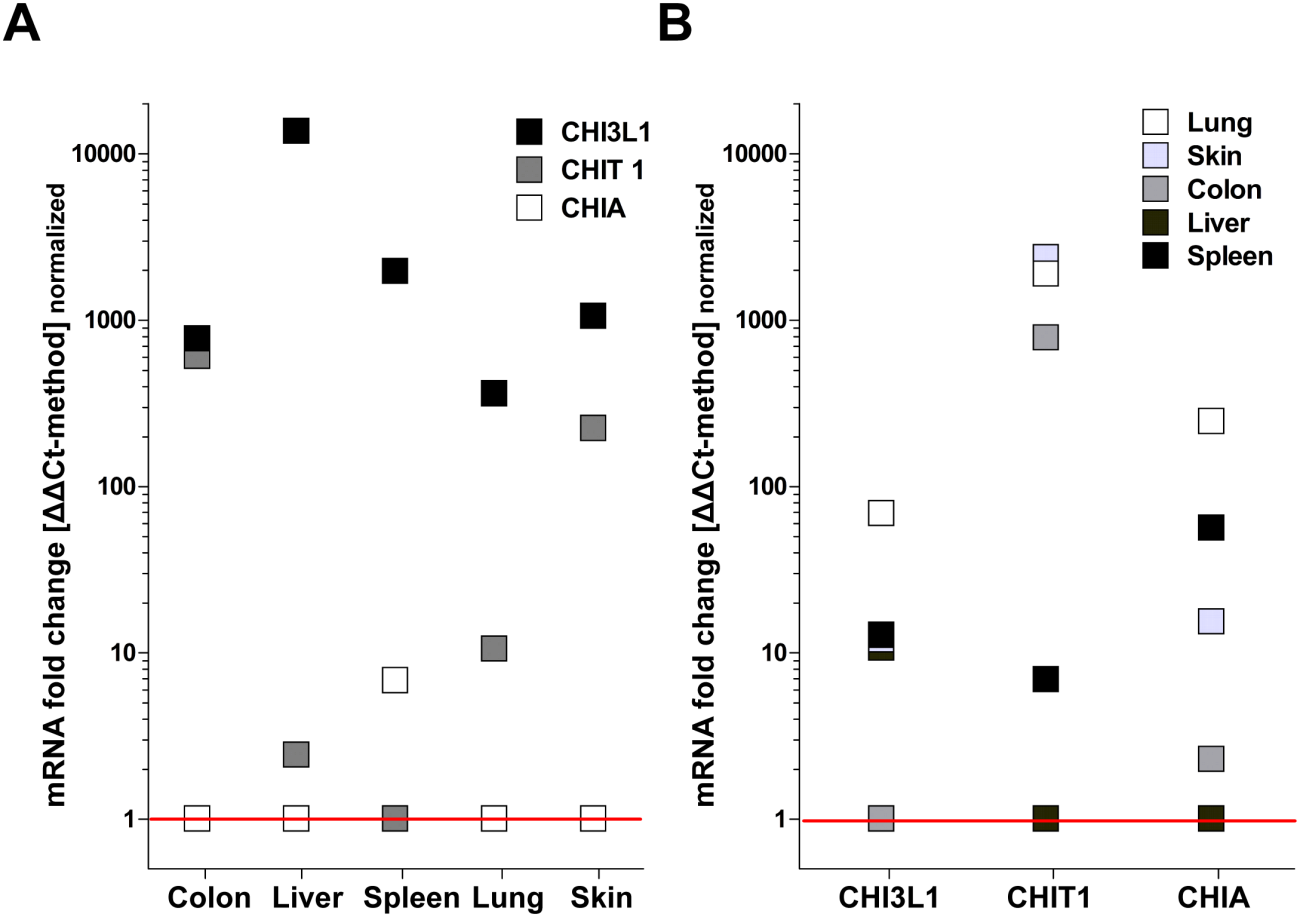
CHI3L1, CHIA and CHIT show distinct, organ-specific expression levels in wild type (WT) mice. (A) Intra-organ comparison regarding skin, spleen, lungs, liver or colon demonstrates pre-dominance of CHI3L1 expression levels over CHIA and CHIT. (B) Inter-organ comparison of CHI3L1, CHIA or CHIT each also reveals an apparent biological relevance e.g. of CHI3L1-expression in lung tissue (qPCR /ΔΔCt-method: n≥4; normalized to lowest expression value of each data set = fold change).

First, we evaluated the organ-specific expression of the three main chitinases found in mammals including humans and WT mice using RT-qPCR: CHI3L1, CHIA and CHIT. Here we distinguished between intra- and inter-organ analysis referring to comparative expression between different chitinases within one organ as well as organ-specific expression levels for each chitinase. The main finding was that CHI3L1 appears to be the predominantly expressed chitinase in mice with ~100–1000 fold higher expression levels compared to CHIA and CHIT in an intra-organ comparison. CHIT1 appeared to be predominently expressed in colon and skin tissue whereas CHIA expression was low in all investigated organs compared to CHI3L1 and CHIA (Fig 1A). Also, with inter-organ analysis, CHI3L1 showed highest constitutive expression levels in lung tissue and lowest in colon conditions. CHIT showed highest expression in lung, skin or colon and lowest in spleen and liver. Again, CHIA showed highest expression levels in lung and lowest in Liver (Fig 1B).

### Modulation of chitinase expression under SIR-conditions in WT mice

**Fig 2:**
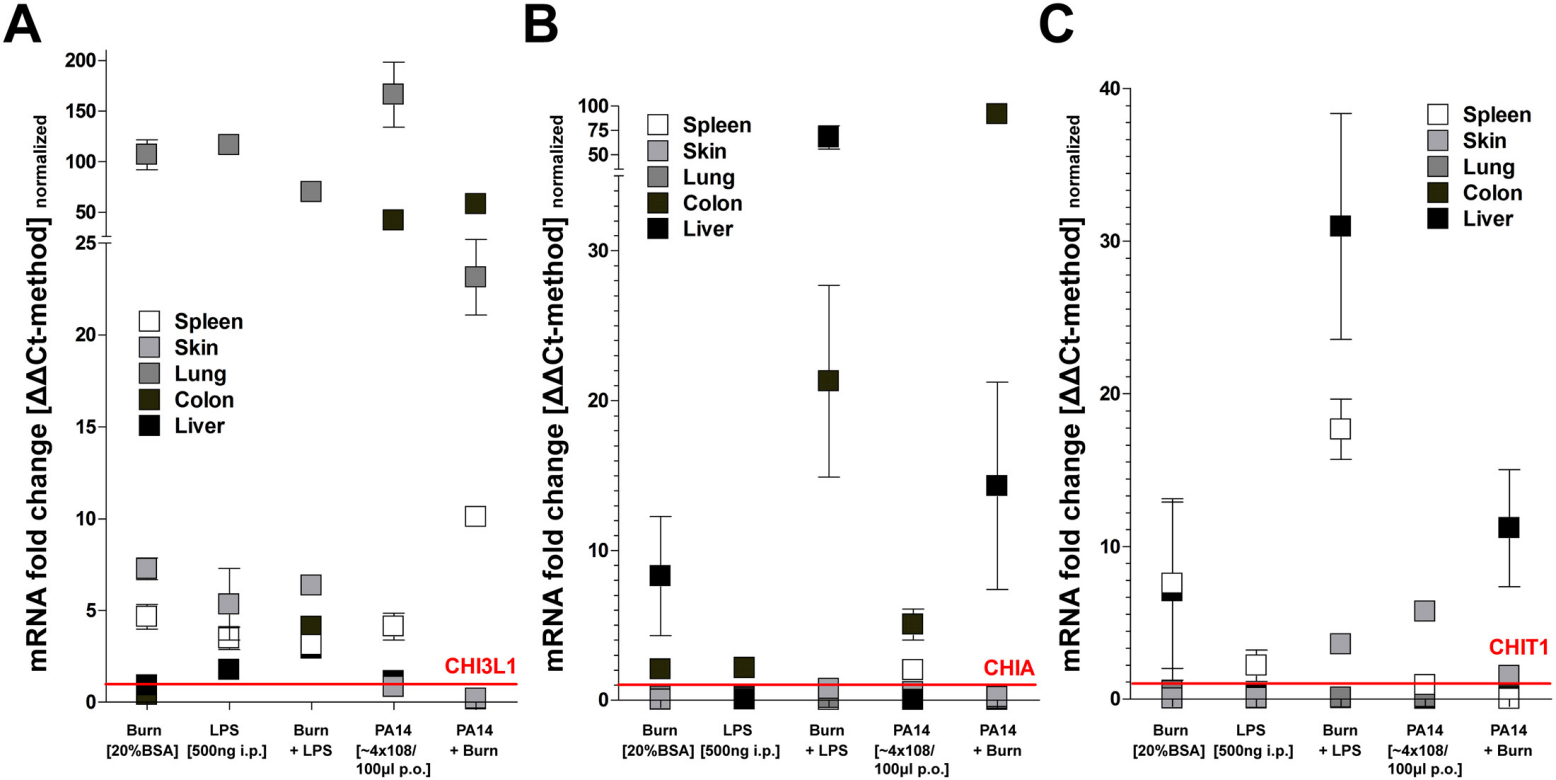
A systemic inflammatory response (SIR) strongly modulates CHI3L1, CHIA and CHIT expression in a mouse model of burn injury and sepsis. WT mice were exposed to burn injury (20% Body Surface Area), lipopolysaccharide (LPS) or oral gavage with Pseudomonas strain (PA14) 48 hours prior to tissue analysis. (A) CHI3L1 expression was strongly induced in lung tissue under all conditions of SIR but also in colon following exposure to PA14 strain. (B) CHIA was predominately induced in liver but also colon following exposure to PA14 strain. (C) CHIT1 appeared to be induced in spleen and liver by burn injury, with or without LPS and PA14 exposure (all: qPCR /ΔΔCt-method: n≥4; mean±SD; normalized to respective organ control).

We continued to compare organ-specific expression levels under pro-inflammatory conditions including burn injury, LPS-induced SIR with/without burn injury or in a burn model of enteric colonization (48h pre-burn) with PA14 Pseudomonas strain. Most notably, CHI3L1 was strongly induced (~100 fold compared to controls) in lung under all pro-inflammatory conditions. Interestingly, colon tissue only responded with an upregulation of CHI3L1 expression following enteric colonization with PA14, further induced with additional burn injury but stayed largely unaffected with burn injury or LPS stimulus. In general, expression of CHI3L1 in liver, skin and spleen was only increased ~5–10 fold, irrespective of the type of pro-inflammatory stimulus (Fig 2A). Conversely, burn injury and LPS, but not PA14 enteric colonization, induced CHIA (~10–100 fold) predominately in colon and liver, but not in lung, skin or spleen. Correspondingly, CHIT 1 was upregulated in liver and spleen, but not lung, skin or colon (Fig 2B/2C).

### Anti-CHI3L1 immunohistolgy (IHC)

**Fig 3:**
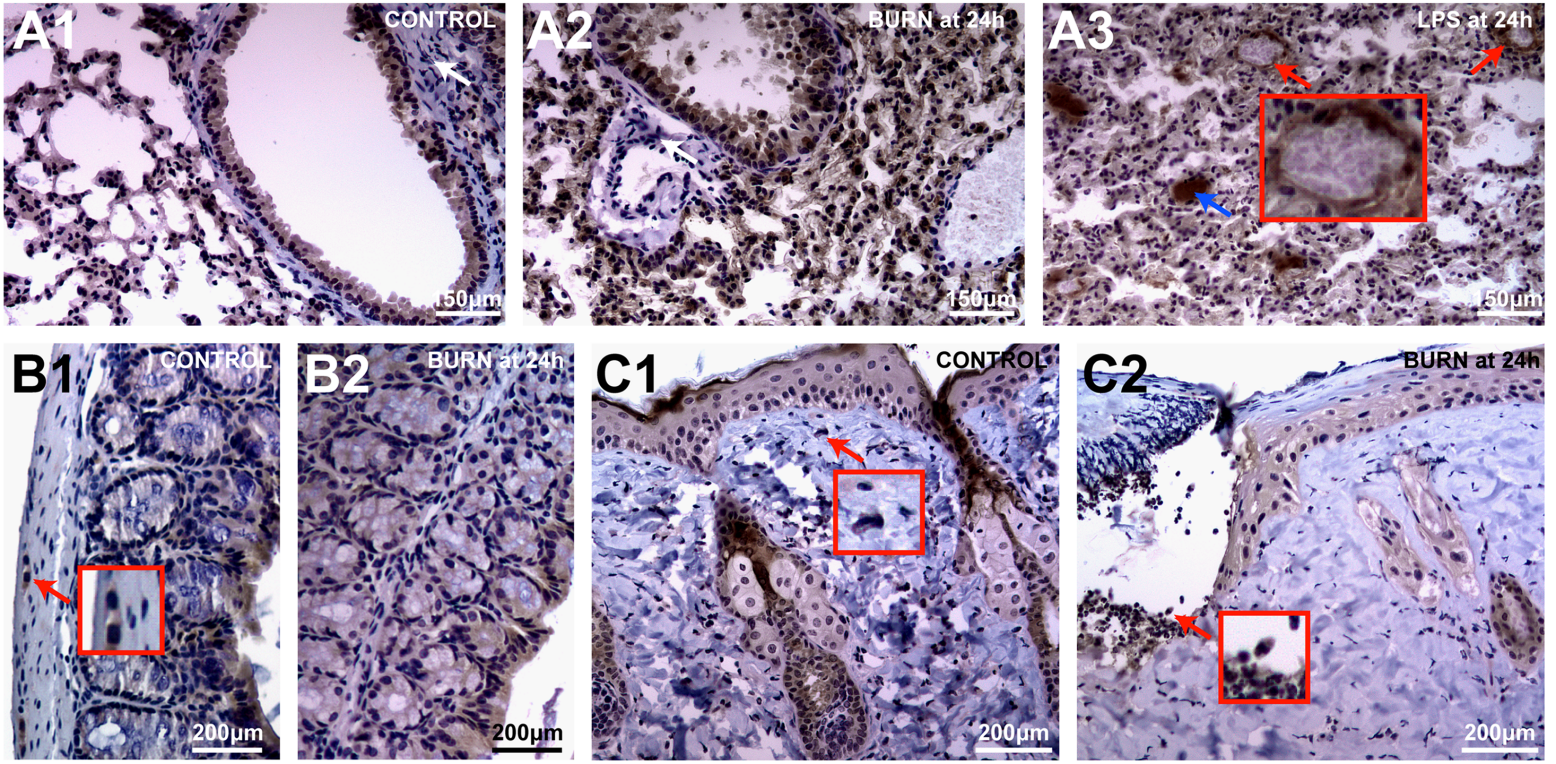
CHI3L1-expression in tissues is dominated by the epithelial lineage as well as a small subset of stromal cells. Immunohistochemistry using anti-CHI3L1 was performed in WT mice. (A1) Both bronchial and alveolar epithelium stain CHI3L1+ in normal lung tissue. (A2) With burn-induced lung injury peri-vascular and–bronchial stromal tissue remains CHI3L1- (white arrow). However, with (A3) severe, LPS-induced SIR, lung tissue demonstrates CHI3L1+ vascular endothelium (red arrow) as well as CHI3L1+ mucous bronchial deposits (blue arrow). (B1) IHC confirms constitutive CHI3L1+ epithelium in colon along with few stromal cells (red arrow) apparently not affected by (B2) burn injury. (C1) Along with epithelium, CHI3L1+ stromal cells are also present in skin (red arrow). (C2) With burn injury, CHI3L1+ granulocytes accumulate at the zone of demarcation.

Based on our transcriptional data, we confirmed CHI3L1 expression using IHC with a focus on representative slides on lung, colon and skin. We identified epithelium as the pre-dominant source of CHI3L1 expression. Though constitutively expressed my bronchial ciliary and alveolar epithelium, CHI3L1-induction in lung appears to be an important feature of SIR following severe burn injury or LPS stimulus (Fig 3A). Notably, with severe lung edema we also saw induction within endothelial vascular lining. As expected, leukocytes homing on lung tissue also stained largely positive for CHI3L1. (Fig 3A3). Conversely, colon appeared to somewhat resistant to pro-inflammatory stimuli (Fig 3B). However, colon epithelial cells appeared to constitutively express CHI3L1 along an apical gradient which progressed to colonic crypts following severe burn injury (Fig 3B1). In addition, an undefined population of large nuclear stromal cells also stained strongly positive for CHI3L1 (Fig 3B1). With the loss of epithelium following full thickness burns, the observed transcriptional upregulation of CHI3L1 in skin was likely due to blood borne innate immune cells homing into the burn wound (Fig 3C2). However, in unaffected skin there appeared to be a small population of resident stromal cells which also stained CHI3L1+ (Fig 3C1).

In conclusion, our findings so far indicated a significant modulation of chitinase expression, especially of CHI3L1, in the context of SIR.

### Modulation of chitinase tissue expression using chitosan, a poly-D-glucosamine derivative

**Fig 4:**
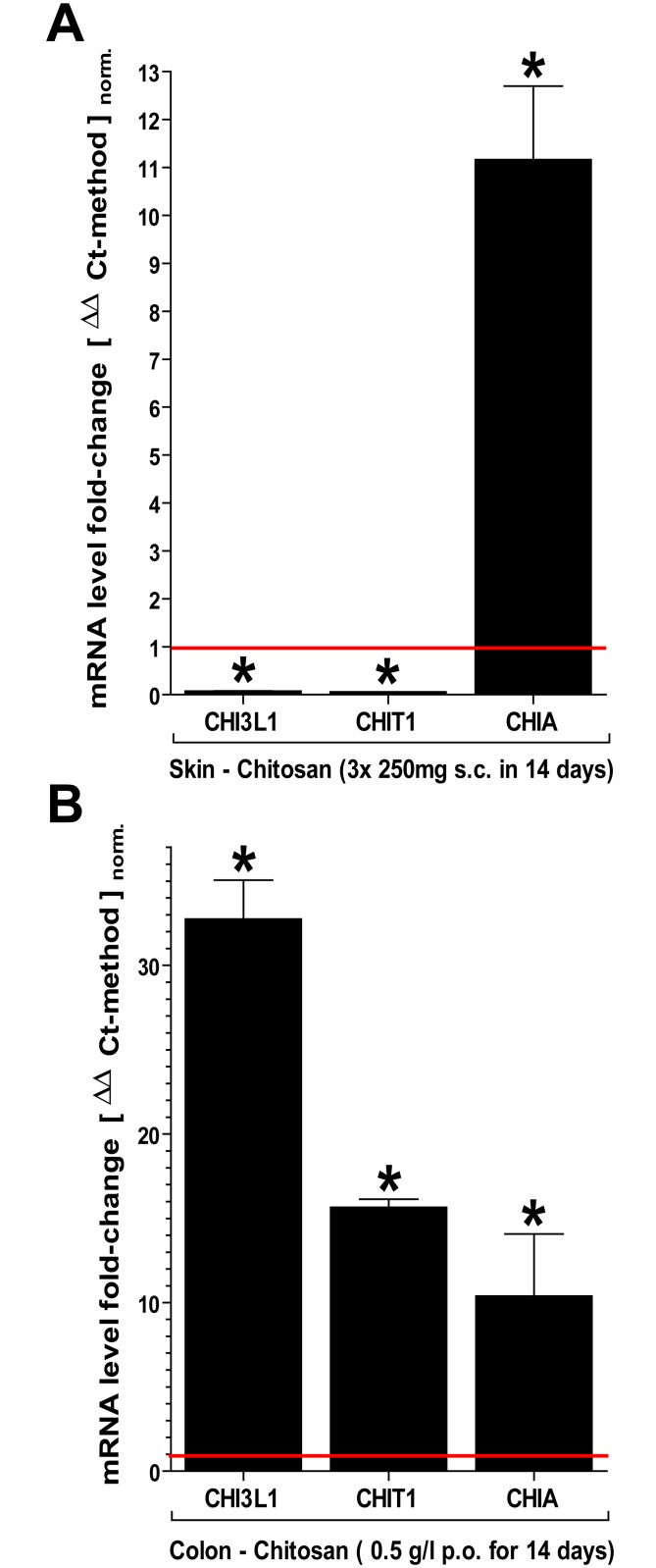
Exposure to chitosan induces chitinase expression in skin and colon. WT mice were exposed to preparations of chitosan (poly-D-glucosamine) s.c. (3x250μg/100μl over 2 weeks) and via drinking water (50mg/l over 2 weeks). (A) With s.c. injection, only CHIA was significantly induced in skin. (B) Chitosan in drinking water induced all chitinases, mostly CHI3L1 (all: qPCR /ΔΔCt-method: n≥4; mean±SD; *P<0.05; normalized to respective organ control).

In order to assess the feasibility of a therapeutic modulation of chitinase expression in burn patients, we exposed WT mice to either s.c. injections or p.o. drinking water containing low molecular weight (lmw) chitosan, a derivative of xenogenic chitin with increased solvability over a prolonged period of time. Both, chitin and chitosan have been comprehensively evaluated under various biomedical applications including wound dressings, nano particles and surface coatings with alleged anti-microbial properties among other [37, 38]. In skin, chitosan induced high expression levels of enzymatically active CHIA but appeared to suppress the expression of CHI3L1 and CHIT1 (Fig 4A). Conversely, in colon, CHI3L1, which showed lowest expression levels under control conditions, was most strongly induced (~30 fold) along with CHIT1 and CHIA (Fig 4B). Thus, chitosan treatment reduced CHI3L1 expression in skin but induced it in colon.

### Modulatory effects of poly-D-glucosamine derivatives on the expression of PA14 Pseudomonas strain virulence factors

**Fig 5:**
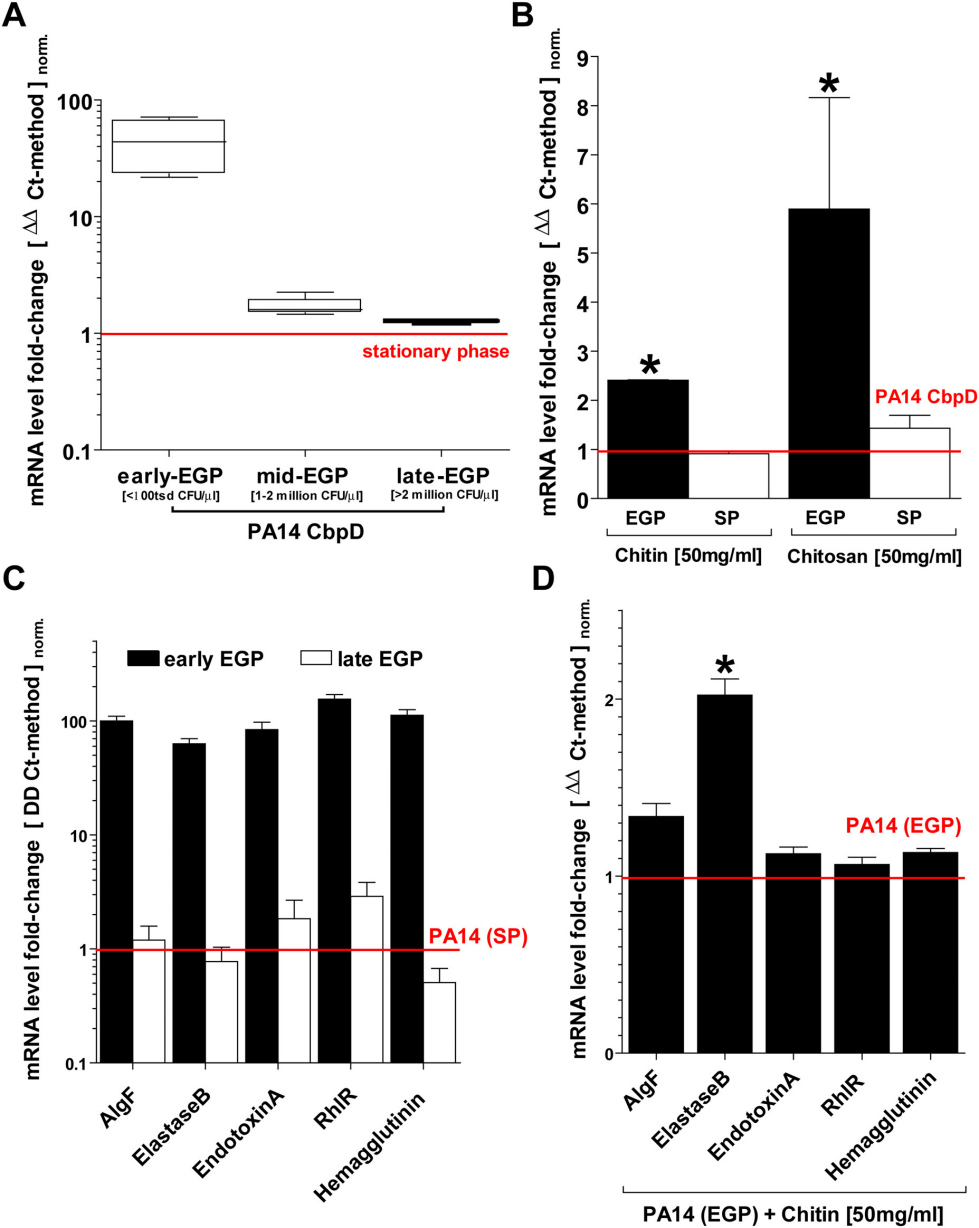
Chitin and chitosan modulate the expression of virulence factors in PA14 Pseudomonas strain. Several factors related to the pathogenicity of Pseudomonas species were evaluated following the addition of chitin (hmv poly-D-glucosamine) and chitosan (lmw poly-D-glucosamine) to lysogeny broth (LB). (A) Chitin-binding-protein Delta (CbpD) is strongly induced early on during the exponential growth phase (EGP) of PA14 strain. (B) Addition of both chitin and chitosan to LB further induces CbpD expression. (C) Various other factors of PA14 pathogenicity are also predominantly expressed during early EGP. (D) However, addition of chitin to LB only further induces ElastaseB but not other virulence factors (all: qPCR /ΔΔCt-method: n≥3; mean±SD; *P<0.05; normalized to a stationary bacterial growth phase control).

The induction of chitinases is related to an innate immune response which might favor infections with pathogens such as PS which can specifically interact with chitinase e.g. through the expression of chitin-binding proteins (CBPs). Mammalian CHI3L1 has been demonstrated as a possible ligand of CbpD along with chitin and chitin derivatives, thus implying a possible role in PA14 virulence. In this context we show that the addition of chitin and chitosan to LB bacterial growth media induces CbpD expression during an exponential growth phase (EGP) (Fig 5A). In addition we saw that low molecular chitosan induced CbpD more effectively than chitin (Fig 5B). During early EGP, PA14 also constitutively expresses various virulence factors related to a clinical relevant infection (Fig 5C). Here, we saw that elastaseB, but no other virulence factors, were specifically induced in PA14 by chitin supplemented growth media, consistent with an environmental ‘sensing-function’ of CbpD during PA14-related opportunistic infection [39] (Fig 5D). Thus, Pseudomonas is likely to specifically alter its transcriptional behavior in a wound environment or epithelial surface in the presence of possible ligands for CbpD such as chitinases, chitin or chitosan.

### Kaplan–Meier estimator of survival rate in a mouse model of opportunistic wound infection with PA14 strain

**Fig 6:**
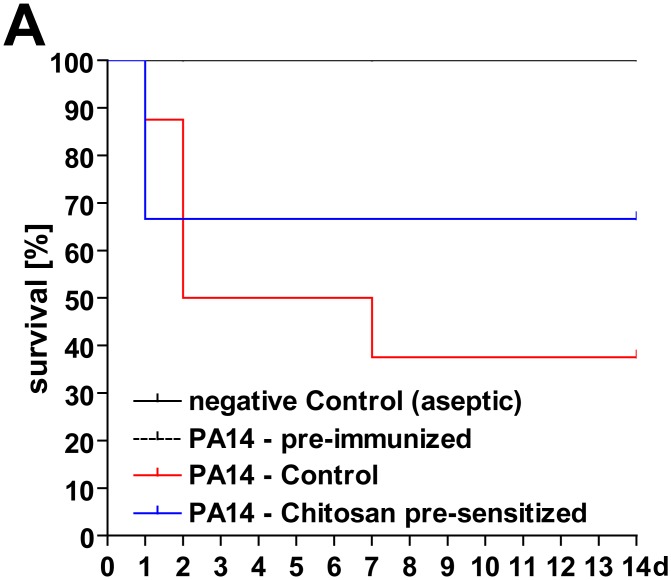
A modulation of chitinase expression in skin increases survival rates in a mouse model of opportunistic wound infection with PA14 strain. Experimental design: (Step 1) WT mice were injected low-dose s.c. with PA14 strain (10^1^ CFU/100μl; see S2 Fig) four weeks prior to experiments in order to induce a uniform PA14-specific immunity. (Step 2) For an additional two weeks animals received s.c.-injections of chitosan (3x250μg/100μl) while the control groups received vehicle in order to induce the expression of chitinases in skin. (Step 3) At 48h post-burn injury (full thickness, dorsal skin fold, ~20% BSA), the burn area was debrided and contaminated with 10^4^ CFU/10μl PA14 strain. We saw that animals pre-sensitized with chitosan were less likely to develop lethal PA14 septicemia (PA14 control v.s. PA14 chitosan-pre-sensitized: n = 10, χ^2^ = 0.31, P = 0.58, hazard ratio = 1.74).

In order to assess the in vivo relevance and possible role of mammalian chitinase expression in facilitating opportunistic wound infection, WT mice were exposed to s.c. injections of chitosan 2 weeks prior to burn injury, thus modulating chitinase expression (also see Fig 4). Following burn injury, debrided burn wounds were contaminated with an optimized dose of PA14 strain (S2A Fig). This model of opportunistic PA14 wound infection among others was characterized by a mortality rate of 100% within 5 days. Also, the burn model demonstrated significantly lower mortality rates if animals were ‘immunized’ to PA14 via enteric colonization several weeks before the experiment (S2A Fig). In this model, we saw a reduced likelihood of fatal PA14 septicemia in animals exposed to chitosan prior to burn injury (Fig 6).

### Kaplan–Meier estimator of survival rate in a mouse model of entero-hematogenic translocation of PA14 strain

**Fig 7:**
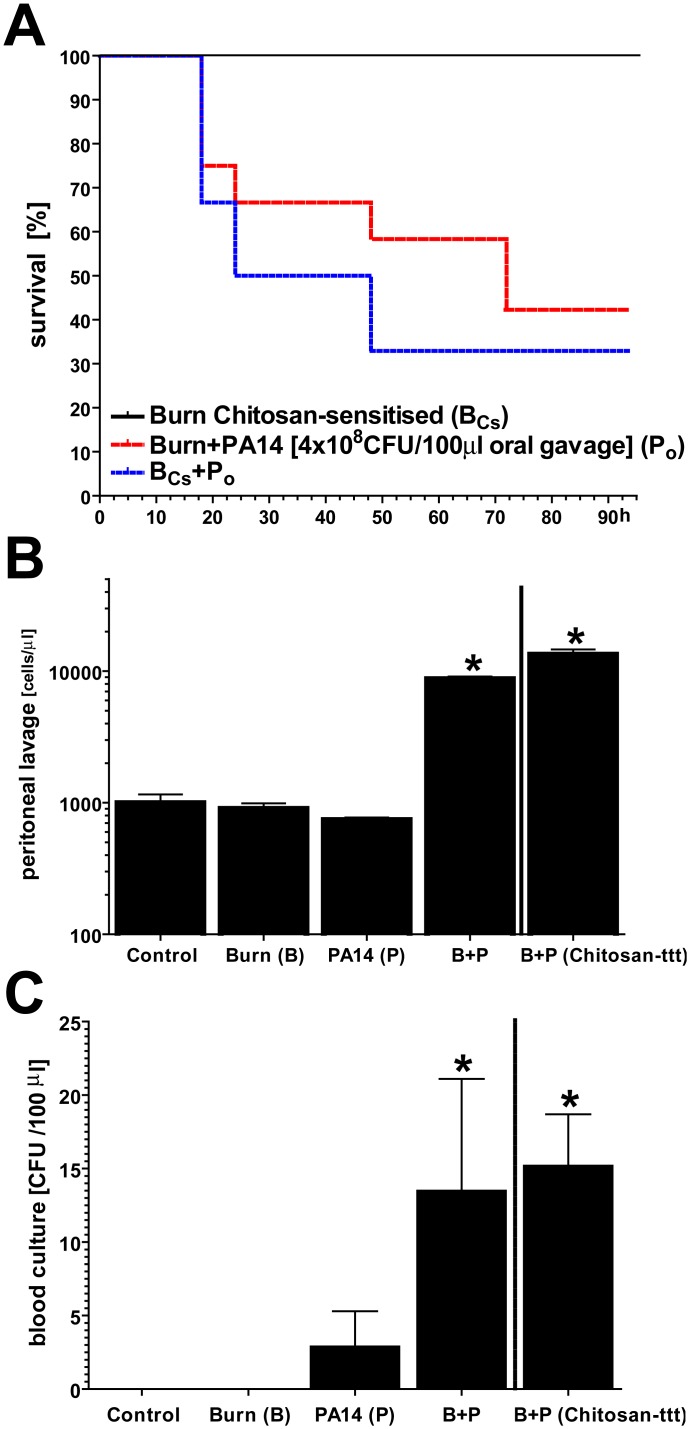
Induced enteric chitinase expression likely promotes entero-hematogenic bacterial translocation following burn injury in a mouse model of enteric colonization with PA14 strain. Experimental design: (Step 1) Acidified drinking water was supplemented with chitosan (50mg/l) four weeks prior to experiments while controls continued to receive unsupplemented acidified water. (Step 2) 48 hours prior to burn injury, enteric colonization with PA14 strain of WT mice was performed (see S3 Fig). (Step 3) With burn injury (full thickness, dorsal skin fold, ~20% BSA), (A) the Kaplan–Meier estimator of survival suggested a higher incidence of lethal PA14 septicemia in animals pre-sensitized with chitosan in drinking water (Burn+PA14 v.s. Burn+PA14+ chitosan-pre-sensitized: n = 12, χ^2^ = 0.39, P = 0.53, hazard ratio = 0.84). (B) Cell count of peritoneal lavage (mostly neutrophils) suggested burn-induced peritonitis due to enteric translocation of PA14 strain, with or without enteric chitinase induction. (C) Similarly, blood culture suggested burn-induced entero-hematogenic translocation of PA14 strain. (B & C: n≥10; mean±SD; *P<0.05; normalized to control)

Increased adhesion, endocytosis and inter-epithelial translocation through the enterocyte-barrier of the intestine have been established as a mechanism of bacterial invasion. We optimized a WT mouse model in which oral gavages with a defined bacterial dose ensured enteric presence of PA14 during the post-burn SIR (S3 Fig). Also in this model, enteric chitinase expression was induced by supplementing chitosan to drinking water (also see Fig 4). We saw that animals with induced enteric chitinase expression prior to burn injury showed mortality rates due to fatal PA14 septicemia of ~70% within 48h opposed to 60% within 72 hours without prior chitosan supplementation of drinking water (Fig 7A). However, group-differences in a leukocyte-count performed on peritoneal lavages (Fig 7B) as well as a CFU-count in blood cultures (Fig 7C) were only significant compared to control but not with ‘therapeutic’ chitosan treatment prior to enteric colonization with PA14. Thus, a net effect of enteric induction of chitinases appears to be a higher likelihood of fatal PA14 septicemia following burn injury although this could not be linked to blood culture results or peritoneal leukocytosis.

### Therapeutic blockage of CHI3L1 using anti-CHI3L1 treatment in a mouse model of PA14 enteric colonization

**Fig 8:**
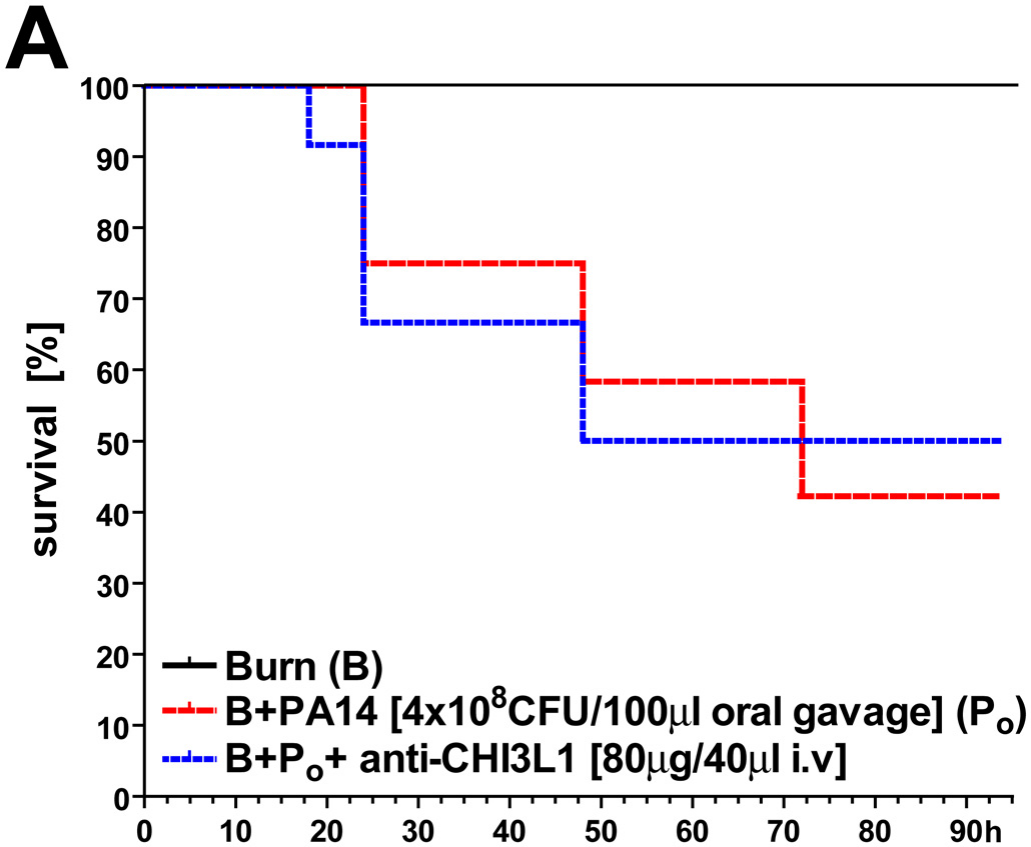
Anti-CHI3L1 pre-treatment did not affect burn-induced PA14 septicemia in a mouse model of PA14 enteric colonization. Experimental design: (Step 1) 72 hours prior to burn injury, enteric PA14 colonization of WT mice was performed (see S3 Fig). 24 hours prior to burn injury, animals received anti-CHI3L1 i.v. treatment in order to block CHI3l1 expression, alleged signaling and bacterial translocation. However, no significant effect on the Kaplan–Meier estimator compared to control was observed.

In order to show a modulation of mammalian chitinase expression as an alternative effective measure to prevent opportunistic infection in the wake of burn injury, we evaluated a WT mouse model in which high-dose anti-CHI3L1 antibody was given simultaneously with burn injury (Fig 8). At least in this model, anti-CHI3L1 treatment did not appear to alter the likelihood of fatal PA14 septicemia due to entero-hematogenic translocation, possibly due to ineffective antibody binding to CHI3L1 expressed on enteric epithelial surfaces.

## Discussion

With severe burn injury and the ensuing hyperacute systemic inflammatory response (SIR), the organism is confronted with (i) an impaired innate immune system, as evidenced by a decrease in neutrophil number and function, as well as (ii) a loss of barrier function which promotes SIR-perpetuation and continued depletion of various other cellular and acellular resources. Thus, due to the pro-inflammatory dynamics in severe burn injury, immuno-modulary approaches may be helpful in improving a given impaired state of the patient’s immune system, for example as was suggested for lipid mediators such as resolvins in a burn mouse model [40, 41].

Skin or intestinal or lung epithelium is a physical barrier that provides primary protection against infection. When this barrier is compromised directly or indirectly following burn injury, pathogens have a direct route to infiltrate the body. Hence, a major port of entry of pathogens is the wound itself. Immediately following a thermal burn, the surface of the burn wound is free of microorganisms. Live threatening bacterial colonization of wound areas with opportunistic bacteria including gram-negative (e.g. Pseudomonas spec., Acinetobacter spec., Enterococcus spec., Escherichia coli spec., etc.) but also gram-positive bacteria (e.g. Staphylococcus aureus spec.) can commonly be seen within 48 hours post-burn despite significant improvements in critical burn care over the last decades. Most of these pathogens are notable for their increasing resistance to a broad spectrum of different antimicrobial agents. They are mostly considered to originate from the patients’ GI and upper respiratory tract but also the hospital environment [42–46].

Increasing evidence supports the notion that inhibition of bacterial translocation into the wound site or thorough the intestinal barrier may be an effective alternative to defer clinical relevant infections of wounds. In this context, the expression of mammalian chitinases (in humans: glycosyl hydrolase family 18) has been reevaluated apart from a purely degrading function of non-mammalian chitin derivatives. Most interestingly, human CHI3L1, also known as human cartilage glycoprotein (HCgp-39) or YKL-40, is a ‘chitinase’ with no enzyme activity, as opposed to chitinase 1 (chitotriosidase) and acidic mammalian chitinase (AMCase), with putative signaling and immune-regulatory functions based on findings that (i) chitinases without enzymatic activity (including CHI3L1) typically act as lectins due to the presence of a preserved carbohydrate-binding domain, and are therefore called “chi-lectins” [47, 48]; (ii) CHI3L1 is strongly expressed by macrophages in the synovial membrane of patients with rheumatoid arthritis (RA), suggesting a potential role as an auto-antigen driving T cell-mediated immune responses in RA [17, 49–52]; (iii) increased circulating levels of CHI3L1 have been reported in the serum of patients with colon cancer [47, 53, 54], inflammatory bowel disease [55] and liver cirrhosis [56] with undetectable levels in serum of healthy individuals [57]; (iv) CHI3L1 secretion is increased by several cell types upon stimulation by inflammatory mediators. CHI3L1 has also been shown to downregulate responses to the same mediators [17], suggesting that CHI3L1 may be part of a feedback control mechanism to regulate the inflammatory response. This also suggests an additional mechanism whereby elevated chitinases may promote infection, namely by decreasing pro-inflammatory responses that help wall off bacteria in the gut and skin. However, the biological function of CHI3L1 remains elusive. CHI3L1 is secreted by various cells in vitro, including activated neutrophils [58], granulocytes, differentiated macrophages [59–61] and colon epithelial cells (CECs) [18, 20].

Other mammalian chitinases also seem to play a pathogenic role in local inflammation. For example, it has been reported that acidic mammalian chitinase (AMCase) and Ym-1 are specifically upregulated in response to Th2-mediated inflammation in the lung [62]. IL-13-mediated induction of AMCase by airway epithelial cells and macrophages may underlie the development of airway hyper-responsiveness and inflammatory cell infiltrate in asthma [62]. In addition it has been demonstrated that bacterial chitin-binding proteins play an important role in the degradation of insoluble chitin [63] and Mizoguchi et al. demonstrated that CHI3L1 enhances bacterial adhesion to CECs through the interaction with bacterial CBP [20]. Thus, CHI3L1 over-expression could enhance the adhesion/invasion of intracellular bacteria into intestinal mucosa lining. In this context, the presence of similar CBPs in related micro-organisms (e.g., S. marcescens, S. olivaceoviridis, P. aeruginosa) suggests a common feature of opportunistic infections such as seen with burn wounds.

Our study shows that: (i) in WT mice CHI3L1 and other chitinases show constitutive organ-specific expression levels which (ii) are highly induced under conditions of SIR, especially in the presence of PA14 Pseudomonas strain, most notably in lung and colon epithelium or within a population of activated innate immune cells homing-in on e.g. deepithelialized wound beds; (iii) chitinase expression can be effectively modulated using chitin derivatives such as chitosan via oral or s.c. route administration whereas CHI3L1 expression was upregulated in colon and downregulated in skin; (iv) expression of CbpD and other virulence factors in PA14 Pseudomonas strain can be modulated using chitin-derivatives. Most relevantly, exposure to chitin derivatives prior to burn injury reduced the likelihood of fatal septicemia in a model of PA14 colonization of wound beds. Conversely, we saw strong evidence for increased mortality rates with chitosan-induced up-regulation of chitinase expression within intestinal mucosa prior to burn injury and with enteric presence of PA14 strain. Thus, chitosan-induced down-regulation of CHI3L1 in skin correlated with decreased mortality rates in a model of PA14 wound colonization, as opposed to increased mortality rates with induced upregulation in colonic-epithelium. These observations are supported by a well established CHI3L1-dependent PS adhesion and translocation through enteric epithelium. Since it is also well established that especially innate immunity is dysfunctional [40, 41] following severe burn injury, we speculate that leukocytes might not be able to effectively lyse more ‘resistant’ bacteria such as PS once internalized, actively promoting septicemia. Known anti-microbial effects of chitin and chitosan, for example when used in wound dressings [37, 38], might also be explained by preventing PS septicemia via leukocyte-dependent translocation. While the administration of anti-CHI3L1 antibody was not therapeutically effective for various possible reasons, a modulation of CHI3L1 expression still appears to be a feasible strategy if applied locally on wound beds but could proof counter-productive if leading to enteric upregulation of CHI3L1, at least during an initial ‘vulnerable phase’ with impaired immunity following burn injury.

Here, a link between CHI3L1-expression and the clinical outcome of opportunistic infection in the context of burn injury could be further established using CHI3L1 knock-out models. The later were no available to the authors at the time. Also, further investigation is warranted regarding possible effects of CHI3L1-expression e.g. on phagocyotic activity and or pro-inflammatory signaling of innate immune cells in the context of CbpD-expressing bacteria.

We conclude, based on the data presented herein, that there is an apparent interaction between CHI3L1 expression on epithelial surfaces or innate immune cells and the ability of CbpD-expressing PA14 Pseudomonas strain to quickly translocate through critical barriers, thus causing septicemia under conditions where innate immunity is temporarily compromised due to overwhelming SIR such as seen in severe burn injury [9–13]. Thus, the idea of a topical but not oral therapy using chitin derivatives might be worthwhile to pursue in order to defer a rapid colonization, raising the likelihood of fatal septicemia emanating from burn wounds. Since a time course of burn wound colonization with multi-resistant Pseudomonas strain can be quite prolonged and is highly problematic in terms of secondary-intent wound closure using bio-active wound dressings or grafts [64] (xenograft, cadaveric skin grafts, autologous skin grafts), a vaccination strategy [65–67] based on CbpD expression might also be feasible as demonstrated by the investigated animal models.

## Supporting Information

S1 Fig Anti-CHI3L1 isotype controls. Please refer to Fig 3. (A) lung tissue, (B) colon tissue, (C) skin tissue.
(TIF)

S2 Fig Kaplan–Meier estimator of survival rate for s.c. injection of a CFU-defined amount of PA14 stock in a mouse model. (A) WT animals were injected s.c. on the dorsal skin fold with colony forming unit (CFU)-defined dosages of PA14 Pseudomonas strain (washed, stock at 4°C, 100μl saline vehicle). Lethal outcomes of ensuing septicemia occurred at 10^5^ CFU count. We defined optimal dosage for outcome studies with 10^7^ CFU count. (B) Both models of excisional and debrided-burn dorsal wounds were evaluated prior to chitinase-modulation experiments. Here, topical contamination of dorsal wounds with PA14 is related to a high mortality rate, especially following burn injury. Interestingly, previous non-lethal infections with PA14 (10^1^ CFU/100μl saline vehicle s.c.) 4 weeks prior to experiments strongly decreased lethality.
(TIF)

S3 Fig Kaplan–Meier estimator of survival rate in a mouse model of burn-induced enteric -derived PA14 septicemia. Experimental design: 48 hours prior to burn injury, enteric colonization of WT mice was achieved using oral gavage with washed PA14 strain (10^8^ CFU/100μl). Compared to non-PA14 colonized animals, this led to highly significant mortality rate likely due to post-burn bacterial entero-hematogenic translocation.
(TIF)
