# Supplementary figures and images for: The Role of CHI3L1 (Chitinase-3-Like-1) in the Pathogenesis of Infections in Burns in a Mouse Model
Source: PLoS One. 2015 Nov 3;10(11):e0140440. doi: 10.1371/journal.pone.0140440 (PMC4631332; doi:10.1371/journal.pone.0140440)

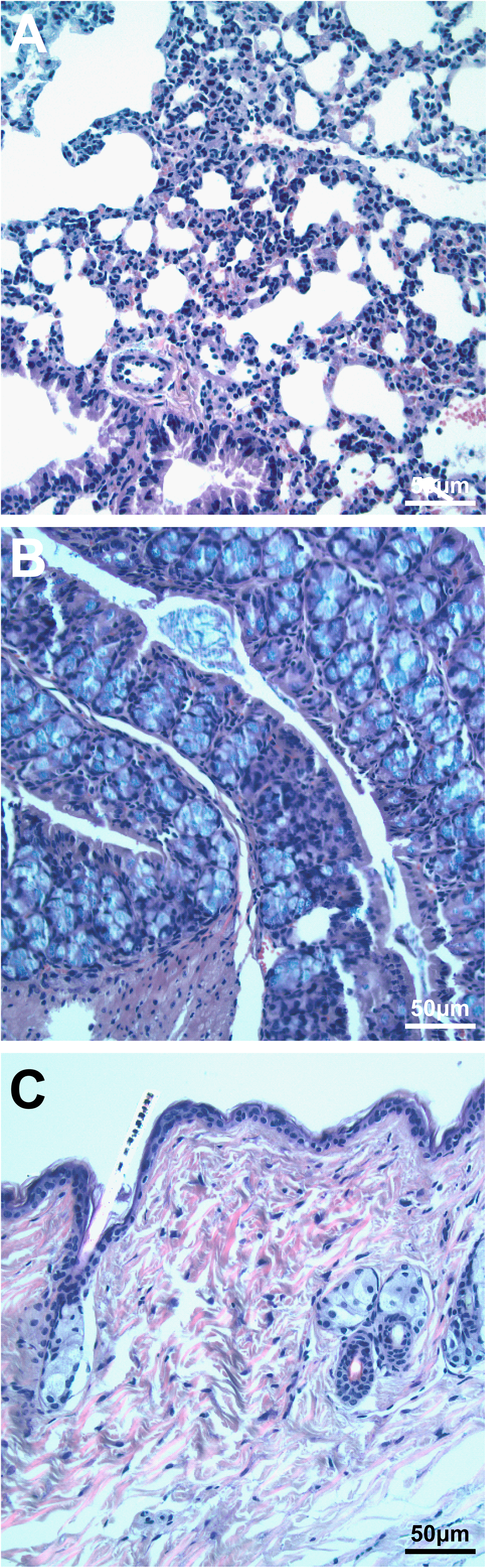

Supplement: S1 Fig — Please refer to Fig 3. (A) lung tissue, (B) colon tissue, (C) skin tissue. (TIF) [file pone.0140440.s001.tif]

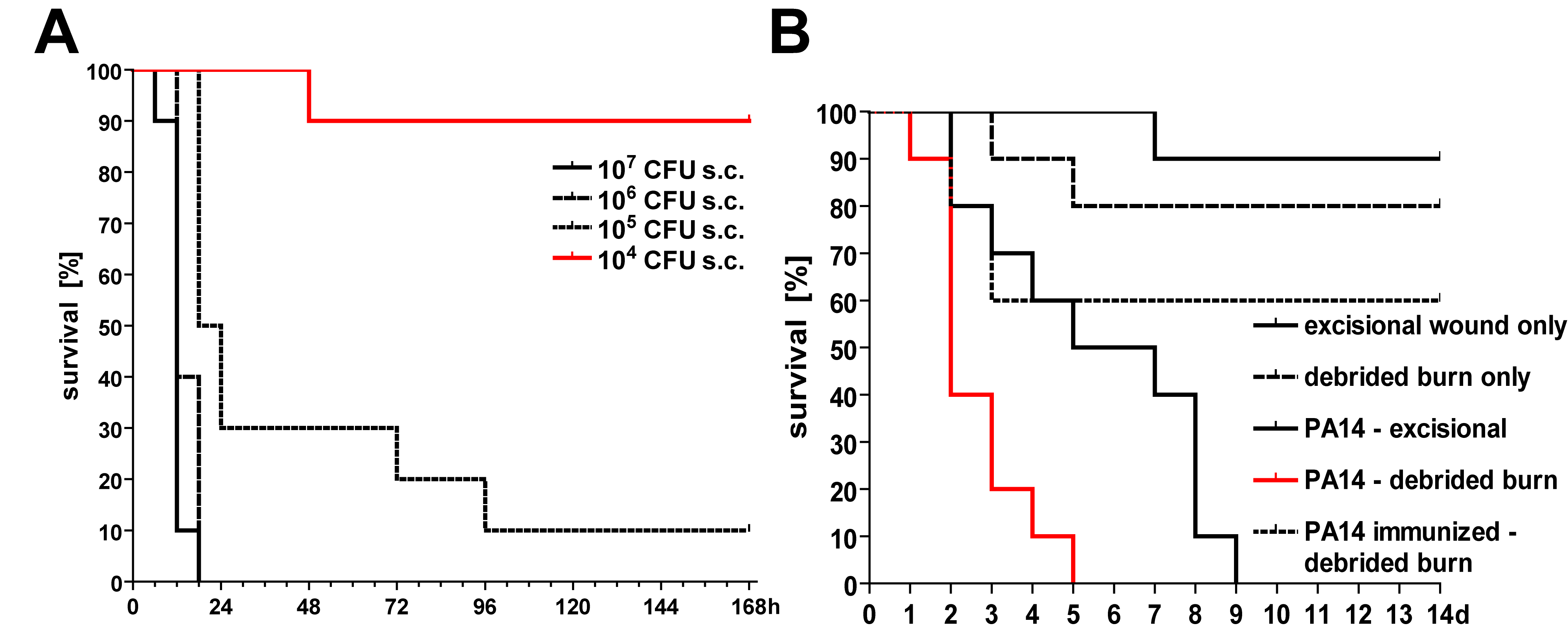

Supplement: S2 Fig — (A) WT animals were injected s.c. on the dorsal skin fold with colony forming unit (CFU)-defined dosages of PA14 Pseudomonas strain (washed, stock at 4°C, 100μl saline vehicle). Lethal outcomes of ensuing septicemia occurred at 105 CFU count. We defined optimal dosage for outcome studies with 107 CFU count. (B) Both models of excisional and debrided-burn dorsal wounds were evaluated prior to chitinase-modulation experiments. Here, topical contamination of dorsal wounds with PA14 is related to a high mortality rate, especially following burn injury. Interestingly, previous non-lethal infections with PA14 (101 CFU/100μl saline vehicle s.c.) 4 weeks prior to experiments strongly decreased lethality. (TIF) [file pone.0140440.s002.tif]

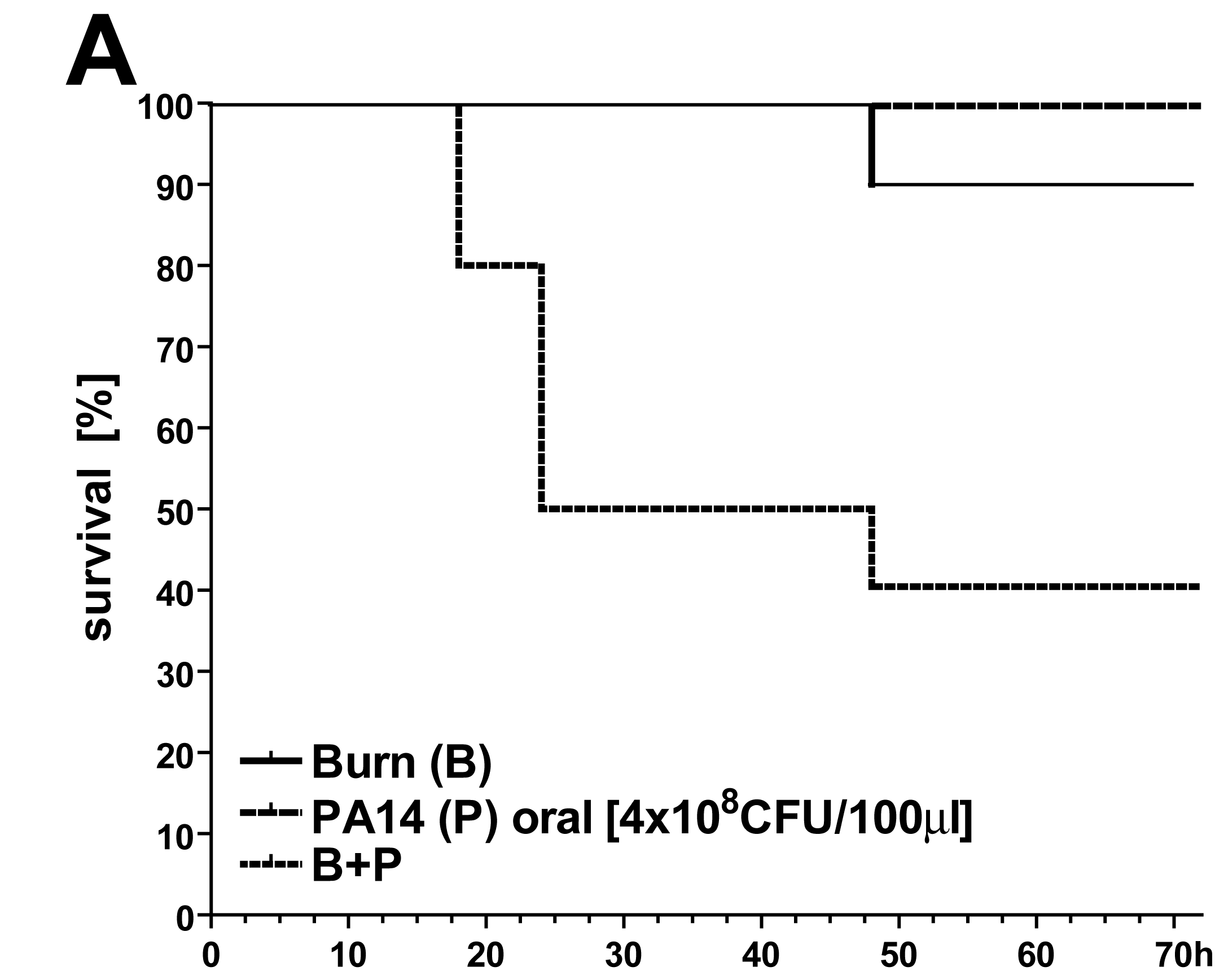

Supplement: S3 Fig — Experimental design: 48 hours prior to burn injury, enteric colonization of WT mice was achieved using oral gavage with washed PA14 strain (108 CFU/100μl). Compared to non-PA14 colonized animals, this led to highly significant mortality rate likely due to post-burn bacterial entero-hematogenic translocation. (TIF) [file pone.0140440.s003.tif]
